# Cambodia: the first national study of antibiotic prescribing and resistance using mixed methods approach

**DOI:** 10.1186/2047-2994-4-S1-P183

**Published:** 2015-06-16

**Authors:** C Om, M-L McLaws, E Vlieghe, F Daily, JC McLaughlin

**Affiliations:** 1 School of Public Health and Community Medicine, UNSW, Sydney, Australia; 2 Department of Clinical Sciences, Institute of Tropical Medicine (ITM), Antwerp, Belgium; 3 Diagnostic Microbiology Development Program, Diagnostic Microbiology Laboratory, Phnom Penh, Cambodia

## Introduction

Antibiotic resistance is present globally. Contributing factors in Cambodia include self-medication by the community, uncontrolled sale of antibiotics and unregulated antibiotic use in food animals.

## Objectives

Our objective was to explore the prescribing practices of physicians in Cambodia and perceptions of antibiotic use and resistance in the country.

## Methods

A sample of 482 physicians from 6 national hospitals was surveyed for knowledge and prescribing practices using common disease scenarios. Focus group discussions (FGD) with physicians explored barriers to evidence-informed prescribing. Approval was given by health research ethics committee of Cambodia, UNSW Australia and ITM.

## Results

Our preliminary findings from combining the survey and FDG results identified that while physicians were aware of antibiotic resistance and perceived prescribing antibiotics appropriately as difficult, they were unlikely to utilize their diagnostic microbiology service to assist their daily prescribing practice. The most common barrier to evidence-based prescribing was their habitual and routine prescribing practices and that of their peers instead of utilizing the microbiology service. Habitual prescribing practices remain unchanged despite the introduction of microbiology services. Surveyed physicians commonly reported that their patients at the time of admission had self-medicated. During discussions physicians reported that antibiotic choice was based on severity of clinical presentation: milder presentations were prescribed ampicillin and gentamicin while severe presentations or patients who had self-medicated were immediately prescribed ceftriaxone. Support from microbiology services was only accessed when patients did not respond to this prescribing regime.

## Conclusion

Antibiotic self-medication and resistance are among challenges for Cambodian physicians to prescribe antibiotics appropriately. Yet, their prescription is likely to be driven by their habit rooted from the era when there was no microbiology laboratory rather than seeking evidence from microbiology laboratory they can now access to. Training programs focusing on rational antibiotic prescribing are urgently needed.

## Disclosure of interest

None declared.

